# Somatotype, body composition, and physical fitness in artistic gymnasts depending on age and preferred event

**DOI:** 10.1371/journal.pone.0211533

**Published:** 2019-02-05

**Authors:** Katarzyna Sterkowicz-Przybycień, Stanisław Sterkowicz, Leon Biskup, Ryszard Żarów, Łukasz Kryst, Mariusz Ozimek

**Affiliations:** 1 Department of Gymnastics and Dance, Institute of Sport Sciences, University of Physical Education, Cracow, Poland; 2 Department of Theory of Sport and Kinesiology, Institute of Sport Sciences, University of Physical Education, Cracow, Poland; 3 Department of Anthropology, Institute of Human Physiology, University of Physical Education, Cracow, Poland; 4 Department of Track end Fields Sports, Institute of Sport Sciences, University of Physical Education, Cracow, Poland; Catholic University of Brasilia, BRAZIL

## Abstract

In men’s artistic gymnastics, results that are particularly appreciated are those obtained in all-around and individual events such as the floor exercise, pommel horse, rings, vault, parallel bars and horizontal bar. However, few studies have explored the dependency of anthropometric characteristics and fitness from age category or the event preferred by gymnasts. Therefore, the aim of this study is to compare the somatic type, body composition and values of some anthropometric and fitness characteristics and indices of gymnasts according to age and preferred event. A total of 53 male gymnasts (19 seniors and 34 juniors) were examined right before the Polish Senior and Junior Championships in Artistic Gymnastics in Warsaw (May 25 to 28, 2017). We examined the characteristics of body length, skeletal system mass, muscle mass, skinfold thickness, and body mass (Tanita S.C.-330S). Body composition (Durnin and Womersley equations), somatotypes (Heath-Carter methodology), handgrip strength (Takei dynamometer), body balance (UPST), the power of the lower limbs (CMJ) were evaluated. Senior gymnasts presented higher than juniors experience, mesomorphy and had higher values in fitness tests of handgrip strength and power of lower limbs (p<0.05). The specialists in floor exercises and vault characterized in higher mesomorphy and lower ectomorphy (p<0.05) and better results of CMJ (p<0.05). We concluded: The seniors demonstrated natural predominance over juniors in several somatic and fitness variables. Detected differences can be useful in the process of identification and development of gymnastic talent. The detected effect of preferred event on certain variables that characterize body build and physical fitness can be useful for choosing a specialization in gymnastic event. A high skill level in all-around events at a national competitive level can be achieved by an athlete characterized by adequate experience, a mesomorphy somatotype component, lower limb index, pelvi-acromial index and relative HGSmax.

## Introduction

The most valued achievements in artistic gymnastics include those obtained in all-around events for six gymnastic events: floor exercises, pommel horse, rings, vault, parallel bars, and horizontal bar [1]. The qualification system has been modified in the Olympic cycle from 2017–2020. The athletes of individual gymnastic events can also qualify for the 2020 Summer Olympics in Tokyo [2]. Anaerobic exercise is typical of these sporting events. The shortest event is the vault (5.16 ± 0.41 s), whereas the longest is floor exercises (60.90 ± 3.44 s). The pommel horse, rings, parallel bars, and horizontal bar performances take approximately 35 s [3]. Overcoming the resistance during hanging exercises and in support requires substantial strength, which impacts the development of the muscular system.

The beauty of this oldest Olympic sport undoubtedly attracts much interest of the spectators. However, practicing this sport at an elite level requires youth [4] and adequate body build for performing gymnastic elements [5]. Studies have evaluated the optimal age to start training [4, 6, 7] and the age of top performance during competitions [4], but the relations of training experience with somatotype and preferred gymnastics event remain unclear.

Previous studies show that gymnasts’ somatotypes are mostly identified as ectomorphic mesomorph (predominantly mesomorphy and ectomorphy rather than endomorphy) and balanced mesomorph (predominantly mesomorphy; endomorphy and ectomorphy are lower and equal or do not differ by more than one-half of a somatotype unit) [8, 9]. The mesomorphy component is more pronounced in athletes with a higher level skill in the sport. Gymnasts selected from the Italian population turn out to have less fat, a more developed mesomorphy component, lower body mass, and lower height compared to non-athletes [5, 10]. This characterization of somatic body build is conducive to higher strength and comprehensive control of the body during gymnastic events. The identification of somatotypes seems to be clear, but there is lack of information about dependency of anthropometric characteristics and indices from age category or events preferred by gymnasts.

There is also insufficient knowledge on the handgrip strength needed for performing technical elements on apparatuses [11] compared to comprehensive analyses performed for grasping sports such as judo [12]. Another important factor in gymnastics is balance [7], especially when landing after dismount from an apparatus in the final phase of routines. Furthermore, substantial power of the lower limbs represents a precondition for fast movement and take-off from the ground, especially during an approach sprint on a runway before a vault or during floor exercises. Optimal anthropometric measurements can ensure higher power for gymnasts. Nevertheless, few studies have analyzed these issues [6, 13].

Nowadays, coaching tendencies for the specialization of athletes in certain gymnastic events are being observed. The training methodology, specific body build characteristics, and physical fitness may make athletes suitable for achieving success in preferred events. Therefore, the aim of this study was to compare gymnasts’ (1) experience, frequency, and time of training, as well as frequency of participation in competitions; and (2) somatotype, body composition, anthropometric indices, and physical fitness according to age and preferred event. These relationships could be used to develop a model of elite gymnasts with a high level of achievement in all-around events. The following research hypotheses were formulated:

H1. The age category of gymnasts influence the intergroup differences in training experience, anthropometric characteristics and indices, somatotype, balance skills, handgrip strength, flight time, height of rise of the center of gravity, and power generated during a counter-movement jump.
H2. There are the intergroup differences in training experience, anthropometric characteristics and indices, somatotype, balance skills, handgrip strength, flight time, height of rise of the center of gravity, and power generated during a counter-movement jump among gymnasts that prefer different events.
H3. There is an effect of interaction between age category and preferred event on some anthropometric and motor characteristics of a sample of Polish gymnasts.
H4. Some combinations of variables that characterize somatic body build and physical fitness are conducive to the achievement of champion skill level in all-around events.

## Material and methods

### Study participants

The study examined 53 male artistic gymnasts were in a competitive period. The gymnasts were divided according to age as seniors (n = 19, age: 21.3 ± 2.62 years, from 17.3 to 25.3 years), who compete at the international (n = 3; [1]) and national (n = 16) levels, and juniors (n = 34, age 14.3 ± 2.15 years, from 8.83 to 17.3 years), who compete at the national (n = 29) and regional levels (n = 5). The procedure of age category division was conducted adequately to the actual declaration of gymnasts and their score-classification in Senior and Junior Championships in Artistic Gymnastics in Warsaw (May 25 to 28, 2017) in senior and junior group respectively. The participants were examined after at least 2 hours following a light meal. The project No. 100/BS/IS/2016 was approved by the Bioethics Committee at the Regional Medical Chamber in Cracow, Poland (No. 215/KBL/OIL/2016 as of 12 December 2016). All of the adult gymnasts signed written consent forms to participate in the study. Consent forms for participation by subjects younger than 18 years old were signed by their parents. We also obtained consent from participants’ coaches for the examinations during the competitive period.

### Procedure

#### Standardized interview

Each participant answered questionnaire items concerning their date of birth, training experience, number of training sessions per week, duration of an individual training session, preferred gymnastic event (-the event that athlete specializes in and obtains the highest scores during competition, the opinion of coach was also taken into account), number of competitions over the year, and greatest achievements during competitions in the last year. Preferred event was also updated based on the participants’ performance scores during the Polish Senior and Junior Championships in Gymnastics (Warsaw, 25–28, May, 2017) [14], before which measurements were performed.

#### Anthropometric measurements

Anthropometric measurements were performed using the Martin’s and Saller’s technique [15]. The following anthropometric devices were used (SIEBER HEGNER MACHINES SA, Switzerland): an anthropometer (accuracy: 1 mm), large and small yoke compasses (accuracy: 1 mm), and anthropometric tape (accuracy: 5 mm). Measurements were also taken using an Alumet dial skinfold caliper with a constant pressure of 10 g/mm^2^ (accuracy: 0.5 mm) and a Tanita SC-330S body composition analyzer. Measurements were taken of heights (body height v, suprasternale sst, acromion a, dactylion da, symphysion sy), widths (shoulder width a-a, pelvis width ic-ic, humerus width cm-cl, femur width epl-epm), and body circumferences (arm flexed and relaxed and arm flexed and tensed, biggest girths of the forearm and calf). The thickness of skinfolds was also measured in the following locations: biceps, triceps, subscapular, abdominal, suprailiac, calf, and thigh. Measurements were also obtained for mass (accuracy of 0.01 kg) and body composition [16]. The measurements were performed by an anthropologist with many years of experience in this field.

The body mass index (BMI), and fat percentage of the whole body mass (PF%) were calculated [16]. Analogously to the BMI (body mass[kg]/body height[m]^2^) [17], we computed the fat-free mass index (FFMI) and fat mass index (FMI) [18]. The results were illustrated in somatograms and in the Hattori body composition chart to compare the values of BMI, FFMI, FMI, and PF% using the same plane. Furthermore, the following anthropometric indices were computed based on body measurements [15]:

upperlimbindex=[(acromionheight‑dactylionheight)/(bodyheight)]x100 (1)

lowerlimbindex=[symphysionheight/bodyheight]x100 (2)

intermembralindex=[(acromionheight‑dactylionheight)/symphysionheight]x100 (3)

trunkindex=[(suprasternaleheight‑symphysionheight)/bodyheight]x100 (4)

trunk‑limb‑ratio=[(suprasternaleheight‑symphysionheight)/symphysionheight]x100 (5)

pelvi‑acromialindex=[pelviswidth/acromionwidth]x100. (6)

The somatotypes of the athletes were determined using the Heath–Carter Anthropometric Somatotype Manual [8,19]. Dedicated software [20] was used to compute the mean values and measures of age variability, HWR, endomorphy, mesomorphy, and ectomorphy somatotype components. The somatotypes were determined for each participant and illustrated in somatograms. Comparisons between sub-groups were carried out using a special ANOVA that considers the somatotype attitudinal distances.

#### Fitness testing

Handgrip strength (HGS) was measured using a Takei digital grip strength dynamometer (T.K.K. 5401, Japan), which has an option to adjust the grip to the palm size. The measurements were performed in a standing position with the upper limb kept along the body. After adjustment of the grip, the participant performed one test of HGSmax (in kgf) and a test of the perceived 50% level of maximal grip force (kgf) to examine force sensibility without visual control from the dynamometer’s indications. The test was performed with the dominant hand. There were 48 right-handed subjects and 5 left-handed subjects. The results were analyzed relative to body mass (kgf/kg), and the difference between the obtained and computed force of **½**HGSmax was expressed in kgf and percentages:

delta%=[HGSdiff/½HGSmax]x100 (7)

where HGSdiff denotes the difference between the obtained and calculated ½HGSmax, and **½**HGSmax is the theoretical value. Similar measurements were used previously for judo competitors and a control group [12].

#### Postural balance

An unipedal stance test (UPST) was performed similarly to previous investigations [21, 22]. Following the guidelines, measurements were taken of the time of maintaining the body one-leg standing position with the upper extremities crossed over the chest. The measurements were recorded in seconds using a stop-watch. The foot of the raised leg was to be kept in contact with the supporting leg by touching it just over the medial part of the ankle. Each participant performed the test three times with his eyes closed. The mean of three attempts was calculated, and the best trial was also recorded [23].

#### Power of the lower limbs

The power of the lower limbs was evaluated based on the CMJ without arm swing (Opto Jump Next Modular, Microgate, Italy). The flight time (s) and height (cm) were evaluated. The power was computed based on the following formula [24]:

PeakPower(W)=60.7×(jumphight[cm])+45.3×(bodymass[kg])‑2055. (8)

The power relative to body mass was also computed (W/kg).

### Statistics

The data are presented as the means and standard deviations (SD). Two-way ANOVA was used. Since there was no non-normality of other data (standardized skewness or kurtosis did not fall within the range of -2 to 2) or homogeneity of variance (Bartlett test), the Rank Transform test was used to compare means. The test is a nonparametric equivalent of a two-way ANOVA and is used to calculate the F statistic based on the rank [25, 26]. When an insignificant effect of interaction was found, a simplified model with removed interaction was used. The least significance difference (LSD) was used for post hoc comparisons. The values in tables are presented as means and SD standard deviations or medians (*Mdn*) and interquartile ranges (*IQR*, *25% to 75%*). The level of significance for all tests was set at p ≤ 0.05.

For significantly different variables, the eta-squared effect size (η^2^) was computed and interpreted as follows: 0.01: small; 0.06: medium; 0.14: large [27]). Two factors were analyzed: age category (senior; junior) and a preferred event, as suggested previously [28]: 1) pommel horse (PH, n = 8) (exercises performed only in support), senior (n = 4), junior (n = 4); 2) horizontal bar (HB), parallel bars (PB), rings (SR) (exercises performed in hanging or support, n = 11), senior (n = 5), junior (n = 6); 3) floor exercises (FX) and vault (VT) (where dominant regime is shock interaction with support, n = 12), senior (n = 4), junior (n = 8); 4) all- round events (AA, n = 22), senior (n = 6), junior (n = 16).

The first ten of all-around athletes from the Polish Senior and Junior Championships [14] were used as a model group. Keeping in mind a restrictive assumption needed for the discriminant function, the nonparametric Probabilistic Neural Networks (PNN) method was used [29]. Based on the optimal PNN model, we developed a classification table and computed the odds ratio for accurate classification for the group of top ten athletes with reference to the rest of the group. Statgraphics Centurion XVII software was also employed.

## Results

### Experience, time of training, and frequency of participation in competition

The levels of gymnast experience, frequency and time of training, and frequency of participation in competitions are presented in Table 1. The mean, SD, the median and IQR values are showed. There was an insignificant interaction of age category and preferred event with gymnast experience. An effect of age category was found (F_(1,48)_ = 86.78, p<0.001, η^2^ = 0.564). The mean for seniors was significantly higher than that for juniors. Furthermore, the effect of preferred event was significant (F_(3,48)_ = 3.02, p = 0.039, η^2^ = 0.059). The athletes who preferred the second event group (HB, PB, SR) and Group 3 (VT, FX) had greater experience in the sport than those in Group 4 (all-around), which was homogeneous to Group 1 (pommel horse) (Fisher’s 95% LSD criterion).

**Table 1:** Descriptive statistics of gymnasts experience, frequency and time of training, and frequency of participation in competition.

Item	Senior		Junior		Total	
**Experience (years)**	14.3^a^	2.91	7.8	2.17	10.10	3.99
**1 (PH)**	13.0	3.37	8.8	0.96	10.9	3.23
**2 (HB, PB, SR)**	16.2	2.39	8.4	1.69	12.0	4.50
**3 (FX, VT)**	14.0	4.24	9.3	1.04	10.8	3.33
**4**^b^ **(AA)**	13.8	1.72	6.6	2.36	8.60	3.96
**Age at start (years)** *Mdn*	7.11	2.16	6.76	0.90	6.81	1.30
**1 (PH)**	7.65	0.65	6.23	1.70	7.22	1.42
**2 (HB, PB, SR)**	6.81	1.33	6.67	0.83	6.71	1.58
**3 (FX, VT)**	8.20	1.54	6.86	0.53	6.91	1.11
**4 (AA)**	6.11	1.49	6.81	1.72	6.72	1.75
**Frequency of training per week** *Mdn*	6.0	4.0	6.0	0.0	6.0	0.0
**1 (PH)**	6.0	2.0	6.0	2.0	6.0	2.0
**2 (HB, PB, SR)**	6.0	0.0	6.5	2.0	6.0	1.0
**3 (FX, VT)**	6.0	2.0	6.0	0.0	6.0	0.0
**4 (AA)**	8.0	4.0	6.0	0.0	6.0	0.0
**Competition per year** *Mdn*	3.0^a^	2.0	4.5	3.0	4.0	3.0
**1**^c^ **(PH)**	3.5	1.5	3.0	0.5	3.0	1.0
**2**^d^ **(HB, PB, SR)**	4.0	0.0	4.5	3.0	4.0	2.0
**3 (FX, VT)**	2.5	1.0	3.5	2.5	3.0	2.0
**4 (AA)**	3.5	5.0	5.5	3.0	5.0	4.0

^a^ = different from junior group

^b^ = different from 2 and 3 groups

^c^ = different from group 4

^d^ = different from group 3.

1 (PH) = Pommel horse, 2 (HB, PB, SR) = Horizontal bar, parallel bars, rings, 3 (FX, VT) = Floor exercises, vault, 4 (AA) = All-around.

The number of competitions per year also showed an insignificant effect of interaction. There were effects of the age category (F_(1,48)_ = 4.26, p = 0.045, η^2^ = 0.061) and preferred event (F_(3,48)_ = 5.28, p = 0.003, η^2^ = 0.228). Seniors (mean rank of 21.2) participated in fewer competitions than juniors (mean rank of 30.2). Gymnasts who preferred all-around events (mean rank of 32.3) participated in more competitions per year than those from Group 1 (18.3) and Group 3 (15.7). Group 3 took part in competitions less frequently than Group 2 (mean rank -29.7). The pairs of preferred events 1–2, 1–3, and 2–4 were homogeneous based on the multiple range test (Fisher’s 95% LSD).

For the age of beginning gymnastic training, insignificant effects were found for interaction between age category and preferred event. Insignificant effects of interaction, age category, and preferred event were also found for the frequency of training per week.

### Age, height, weight and somatotype components according to age category and preferred event

In Table 2 an age, height, weight and somatotype components of gymnasts are showed. The mean, SD, the median and IQR values are displayed.

**Table 2:** Descriptive statistics of age, height, weight and somatotype components of gymnasts.

Item	Senior		Junior		Total	
**Age (years)**	21.3^a^	2.62	14.3	2.15	16.8	4.10
**1 (PH)**	20.7	3.18	14.8	0.98	17.7	3.83
**2 (HB, PB, SR)**	22.9	2.07	14.9	1.27	18.5	4.44
**3 (FX, VT)**	21.8	3.55	15.7	1.43	17.7	3.70
**4 (AA)**	20.2	1.81	13.3	2.52	15.2	3.89
**Height (cm)** *Mdn*	170^a^	4.5	164	17.3	169	11.6
**1 (PH)**	176	16.1	167	9.85	169	10.9
**2 (HB, PB, SR)**	170	1.1	166	23.1	170	17.5
**3 (FX, VT)**	168	5.3	168	8.85	168	7.55
**4 (AA)**	171	7.7	161	28.2	164	25.3
**Weight (kg)** *Mdn*	68.3^a^	7.9	54.30	22.8	61.7	17.0
**1 (PH)**	67.0	16.4	54.80	9.7	60.3	12.2
**2 (HB, PB, SR)**	71.0	3.3	56.10	25.3	64.2	20.8
**3 (FX, VT)**	66.0	2.3	62.80	8.4	64.8	6.2
**4 (AA)**	68.8	12.5	42.90	26.2	54.3	27.5
**Endo** *Mdn*	1.9	0.7	1.6	0.5	1.7	0.5
**1 (PH)**	1.9	1.1	1.4	0.2	1.5	0.6
**2 (HB, PB, SR)**	2.0	1.4	1.6	0.5	1.9	0.6
**3 (FX, VT)**	2.0	0.8	1.9	0.4	1.9	0.5
**4 (AA)**	1.8	0.6	1.6	0.6	1.6	0.5
**Meso**	6.5^a^	0.96	5.0	1.09	5.5	1.25
**1 (PH)**	5.6	1.06	5.1	0.29	5.4	0.76
**2 (HB, PB, SR)**	6.6	0.93	4.6	0.81	5.5	1.31
**3**^b^ **(FX, VT)**	6.8	0.86	6.1	0.84	6.3	0.87
**4 (AA)**	6.6	0.90	4.5	1.02	5.1	1.37
**Ecto**	2.0^a^	0.74	3.2	1.02	2.8	1.08
**1 (PH)**	2.8	1.00	3.3	0.34	3.0	0.75
**2 (HB, PB, SR)**	1.7	0.43	3.6	0.82	2.7	1.20
**3**^c^ **(FX, VT)**	2.0	0.55	2.3	0.82	2.2	0.73
**4 (AA)**	1.9	0.72	3.5	1.06	3.1	1.20

^a^ = different from juniors

^b^ = group 3 different from 1 and 2 groups, and 4 group

^c^ = group 3 different from 1 and 4 groups.

1 (PH) = Pommel horse, 2 (HB, PB, SR) = Horizontal bar, parallel bars, rings, 3 (FX, VT) = Floor exercises, vault, 4 (AA) = All-around.

There was no effect of interaction for age. An effect of age category was found (F_(1,48)_ = 113.29, p<0.001, η^2^ = 0.560), and the natural predominance of seniors over juniors was confirmed. There was no effect of preferred event on age. A similar pattern of effects was also found for the variance model for other analyzed variables. No interaction was observed for body height. We found an effect of age category (F_(1,48)_ = 6.67, p = 0.013, η^2^ = 0.110), but there was no effect of preferred event. Furthermore, no interaction occurred for body mass. An effect was found for the age category (F_(1,48)_ = 32.01, p<0.001, η^2^ = 0.358), but not for preferred event.

For the endomorphy somatotype component interaction and effects of the age category and preferred event were insignificant. There was no significant interaction for mesomorphy. We found large effects of both age category (F_(1,48)_ = 27.52, p<0.001, η^2^ = 0.309) and preferred event (F_(3,48)_ = 4.38, p = 0.008, η^2^ = 0.147). Seniors were characterized by significantly higher mesomorphy than juniors. Furthermore, significant differences of means were documented between preferred events. The athletes who preferred VT and FX (Group 3) had better results in mesomorphy than those who preferred PH (Group 1) and HB, as well as PB and SR specialists (Group 2). Moreover, they showed predominance over all-around athletes.

An insignificant interaction was found for ectomorphy. Ectomorphy depended on the age category (F_(1,48)_ = 22.42, p<0.001, η^2^ = 0.282) and preferred event (F_(3,48)_ = 2.98, p = 0.040, η^2^ = 0.113). Seniors had a lower level of ectomorphy than juniors. Group 3 showed lower ectomorphy than Group 1 but higher than Group 4. The sub-groups which represent the 1st, 2nd, and 4th preferred events were homogeneous in terms of mean ectomorphy (multiple comparison test 95% LSD).

**Fig 1:**
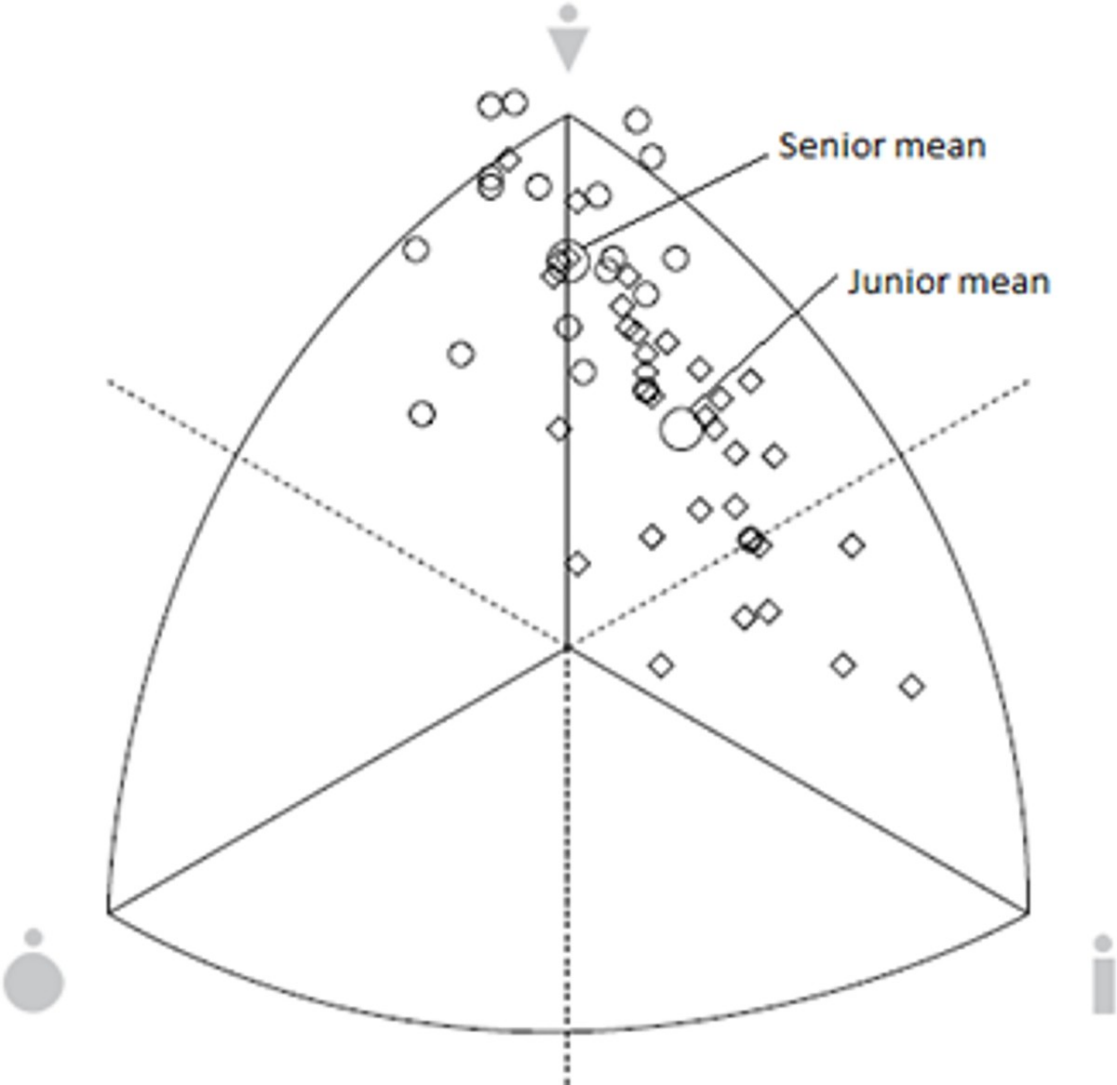
Somatotype distribution. Senior (small circles) and junior (small squares) gymnasts. Large circles = means.

**Fig 2:**
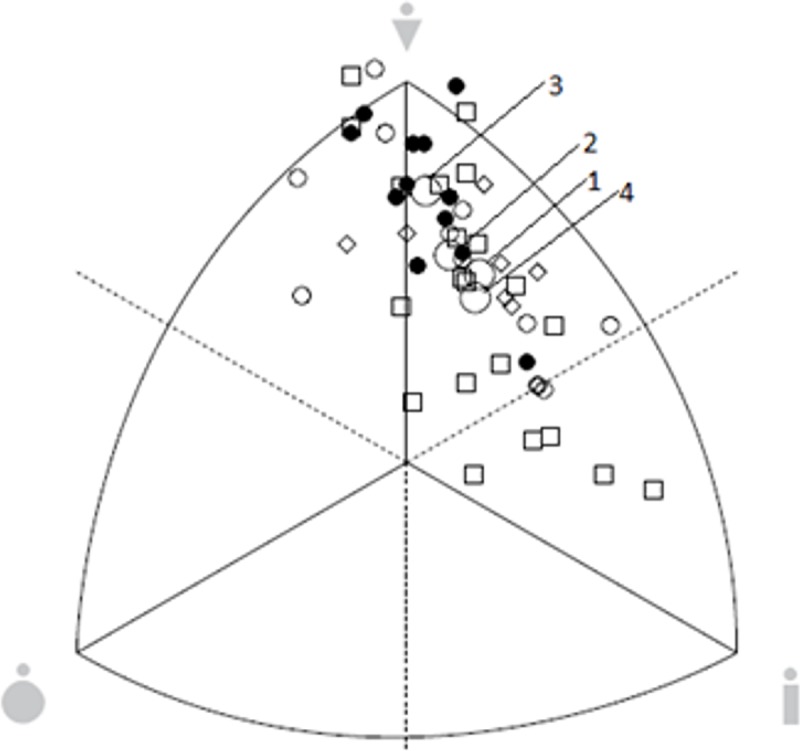
Somatotype distribution for gymnasts who prefer different events. Large circles–means of group: 1 = pommel horse; 2 = horizontal bar, parallel bars, rings; 3 = vault, free exercises; 4 = all-around.

The special ANOVA considering somatotype attitudinal distances revealed a significant effect of age category (F_(1,51)_ = 18.83, p<0.001). The senior group was classified as balanced mesomorphs ($\bar{S}$ = 2.02–6.40–2.04, SD = 0.73–0.96–0.74), whereas participants in the junior group were ectomorphic mesomorphs ($\bar{S}$ = 1.75–4.96–3.23, SD = 0.52–1.09–1.02) (Fig 1). There were significant differences (F = 5.37, p = 0.031) between Group 1 (ectomorphic mesomorph, $\bar{S}$ = 1.71–5.35–3.02, SD = 0.59–0.76–0.75) and Group 3 (balanced mesomorph, $\bar{S}$ = 1.84–6.30–2.19, SD = 0.36–0.87–0.73). Groups 3 and 4 (ectomorphic mesomorph, $\bar{S}$ = 1.83–5.05–3.09, SD = 0.60–1.37–1.20) were also different (F = 6.37, p = 0.016) (Fig 2).

### BMI and body composition variables for male Polish gymnasts according to age category and preferred events

No significant interaction was found for BMI. Effects were observed for age category (F_(1,48)_ = 38.34, p<0.001, η^2^ = 0.390) and preferred event (F_(3,48)_ = 3.22, p = 0.031, η^2^ = 0.097). Seniors showed a higher level (mean rank = 40.6) compared to junior athletes (mean rank = 20.3). Group 3 (mean rank = 38) had better results than Group 1 (mean rank = 26.9) and Group 4 (mean rank = 26) (Table 3).

**Table 3:** Descriptive statistics of BMI and body composition variables for male Polish gymnasts according to age category and preferred event.

Item	Senior		Junior		Total	
**BMI (kg/m**^**2**^**)** *Mdn*	23.7^a^	1.9	19.65	4.3	21.2	11.0
**1 (PH)**	22.0	3.4	20.0	1.2	20.7	2.0
**2 (HB, PB, SR)**	24.4	1.3	19.9	4.1	22.3	4.9
**3**^b^ **(FX, VT)**	23.05	1.35	22.55	3.1	22.85	2.1
**4 (AA)**	23.7	1.1	18.55	4.8	19.35	10.8
**FFM (kg)**	61.5^a^	5.96	46.6	12.40	52.0	12.7
**1 (PH)**	60.9	7.04	52.4	5.47	57.0	7.51
**2 (HB, PB, SR)**	62.4	5.06	54.6	7.50	57.4	7.59
**3 (FX, VT)**	64.9	5.12	42.2	16.70	53.6	16.6
**4 (AA)**	59.1	6.37	40.8	12.90	44.4	13.9
**FFMI (kg/m**^**2**^**)**	21.0 ^a^	1.31	17.8	2.41	18.9	2.59
**1 (PH)**	20.4	1.33	18.3	0.69	19.4	1.52
**2 (HB, PB, SR)**	21.8*	0.90	19.8	1.60	20.5	1.67
**3 (FX, VT)**	21.7	0.53	16.8	2.76	19.3	3.18
**4 (AA)**	20.6*	1.74	16.6	2.45	17.4	2.80
**FM (kg)**	7.19^a^	2.52	4.93	1.81	5.74	2.34
**1 (PH)**	7.86	3.33	4.98	1.18	6.53	2.89
**2 (HB, PB, SR)**	6.58	1.68	6.09	1.45	6.26	1.49
**3 (FX, VT)**	5.27	1.39	3.83	3.06	4.55	2.27
**4 (AA)**	8.23	1.99	4.46	1.75	5.21	2.33
**FMI (kg/m**^**2**^**)**	2.44^a^	0.78	1.88	0.52	2.08	0.68
**1 (PH)**	2.61	0.96	1.72	0.29	2.12	0.83
**2 (HB, PB, SR)**	2.3	0.59	2.20	0.419	2.20	0.97
**3 (FX, VT)**	1.75	0.35	1.47	0.88	2.23	0.49
**4 (AA)**	2.86	0.65	1.83	0.52	1.92	0.53
**PF(%)**	10.4	2.94	9.48	2.26	9.80	2.54
**1 (PH)**	11.2	3.12	8.25	1.28	9.73	2.71
**2 (HB, PB, SR)**	11.6	3.97	8.50	2.56	9.93	3.50
**3 (FX, VT)**	9.68	2.85	9.97	1.49	9.88	1.91
**4 (AA)**	9.23	1.94	9.91	2.57	9.73	2.39

^a^ = different from junior

^b^ = different from 1 and 4 groups

* = interaction effect.

1 (PH) = Pommel horse, 2 (HB, PB, SR) = Horizontal bar, parallel bars, rings, 3 (FX, VT) = Floor exercises, vault, 4 (AA) = All-around.

**Fig 3:**
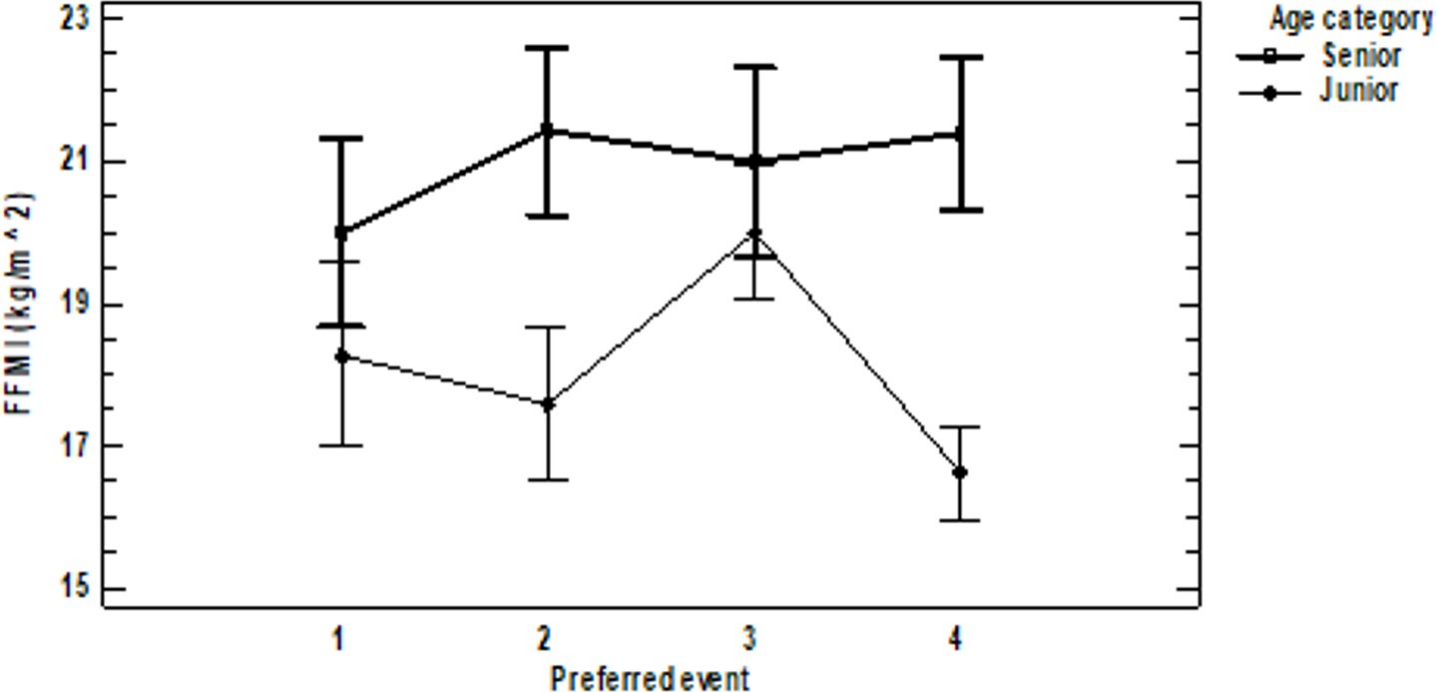
Interactions and 95% LSD for FFMI. FFMI = fat-free mass index.

No interaction was observed for FFM. We found a significant effect of age category (F_(1,48)_ = 22.93, p<0.001, η^2^ = 0.278), but no effect was observed for preferred event. Seniors had higher FFM than juniors. FFMI showed an effect of interaction (F_(3,45)_ = 2.88, p = 0.046, η^2^ = 0.085). The effect of the senior age category was suppressed if the preferred event was Group 2 (HB, PB, SR) and Group 4 (AA) (Fig 3).

**Fig 4:**
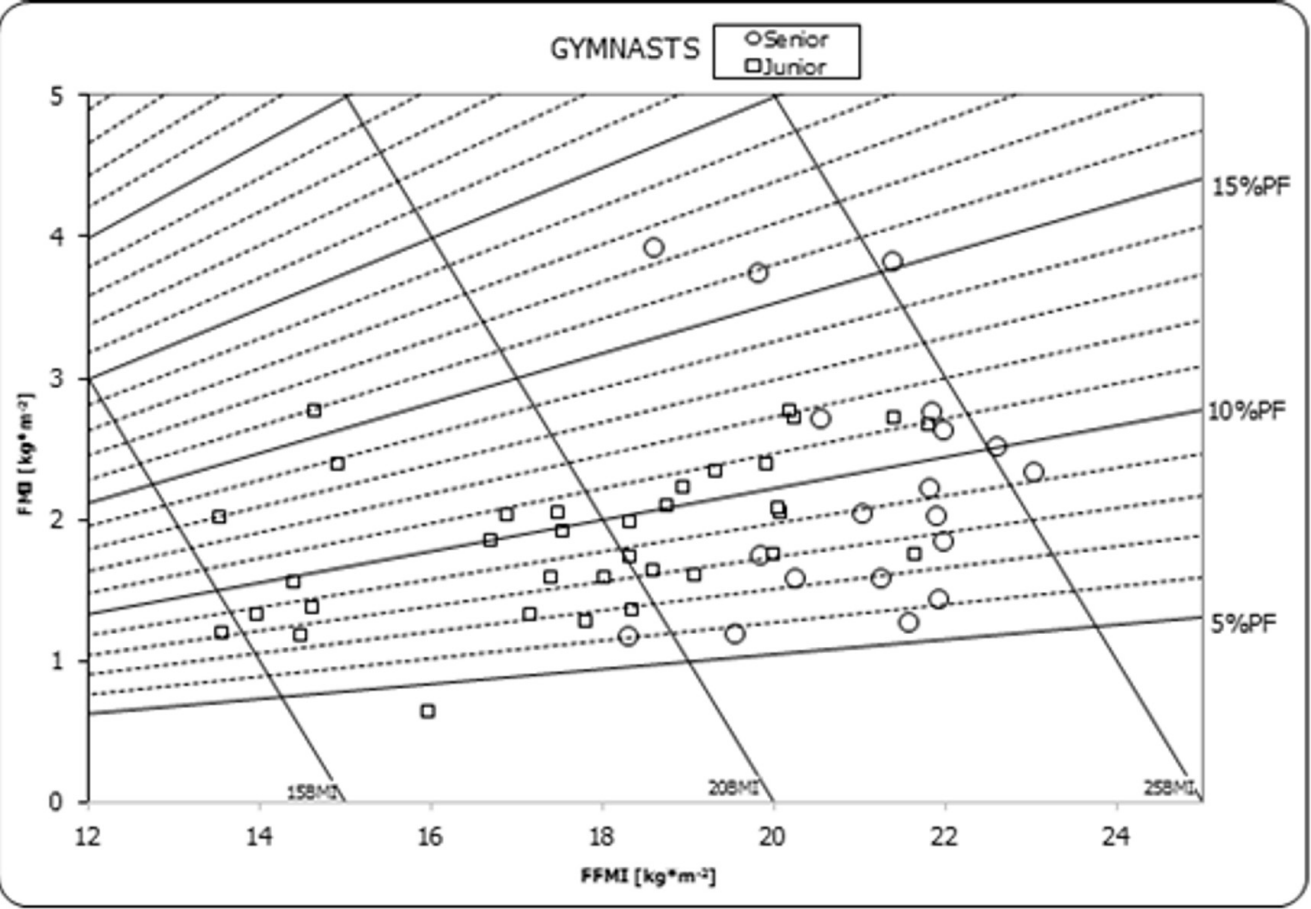
Gymnasts body composition chart. BMI = body mass index, FFMI = fat-free mass index, FMI = fat mas index, PF% = percent fat in whole body mass.

No effect of interaction was found for FM. An effect of the age category was found (F_(1,48)_ = 12.23, p = 0.001, η^2^ = 0.186), but the effect of preferred event was not significant. A similar pattern for FMI means considering the interaction was observed for age category (F_(1,48)_ = 8.85, p<0.005, η^2^ = 0.149) and preferred event. No significant interaction was found between age category and preferred event for PF(%). Furthermore, no main effects were identified for age category and preferred event. The relationships between BMI, FFMI, FMI, and PF(%) are shown in Fig 4.

BMI for seniors ranged from 20.5 to 25.8 kg/m^2^ and was higher than for juniors (14.7 kg/m^2^ to 24.5 kg/m^2^). No underweight was found in junior and senior groups. In the senior group, three individuals were overweight (from 25 to 26 kg/m^2^). Most of gymnasts presented normal BMI values. FFMI ranged from 18.30 to 23.00 kg/m^2^ in seniors and from 13.5 to 21.8 kg/m^2^ in juniors. FMI differed between seniors (from 1.35 to 3.92 kg/m^2^) and juniors (0.64 to 2.76 kg/m^2^).

### Characteristics of length indices and pelvi-acromial index according to age category and preferred event

The upper limb index showed no significant interaction between age category and preferred event, furthermore no main effects were found for age category and preferred event. A similar pattern of ANOVA results was observed for intermembral index and trunk index (Table 4).

**Table 4:** Body length indices and pelvi-acromial index according to age category and preferred event.

Item	Senior		Junior		Total	
**Upper limb index (%)**	43.5	1.27	44.0	1.07	43.8	1.16
**1 (PH)**	43.4	1.40	44.1	1.40	43.7	1.34
**2 (HB, PB, SR)**	43.2	1.54	44.0	1.09	43.6	1.30
**3 (FX, VT)**	43.8	1.54	43.6	0.70	43.7	1.00
**4 (AA)**	43.5	1.07	44.1	1.19	44.0	1.16
**Lower limb index (%)**	51.4^a^	1.26	52.4	1.27	52.1	1.35
**1 (PH)**	52.0	0.86	52.7	0.82	52.3	0.85
**2 (HB, PB, SR)**	50.6	1.63	53.4	1.27	52.1	2.02
**3 (FX, VT)**	51.6	1.18	52.4	1.14	52.1	1.16
**4 (AA)**	51.6	1.14	52.0	1.30	51.9	1.24
**Intermembral index (%)**	84.6	2.46	83.9	2.27	84.1	2.34
**1 (PH)**	83.5	3.67	83.7	3.60	83.6	3.37
**2 (HB, PB, SR)**	85.4	2.68	82.3	1.87	83.7	2.71
**3 (FX, VT)**	84.9	2.03	83.3	2.15	83.8	2.17
**4 (AA)**	84.4	1.95	84.8	1.81	84.7	1.81
**Trunk index (%)** *Mdn*	29.8	1.96	28.9	1.71	29.0	1.75
**1 (PH)**	29.6	2.61	29.0	1.20	29.0	1.90
**2 (HB, PB, SR)**	30.0	2.92	28.4	2.39	28.7	3.17
**3 (FX, VT)**	29.4	1.13	28.6	2.43	29.0	1.98
**4 (AA)**	29.5	2.49	29.1	1.34	29.1	2.02
**Trunk-to-limb ratio (%)**	57.4^a^	4.09	55.0	4.02	55.9	4.17
**1 (PH)**	56.3	4.05	54.4	2.62	55.3	3.31
**2 (HB, PB, SR)**	59.1	6.04	52.5	3.90	55.5	5.84
**3 (FX, VT)**	57.0	2.27	55.1	4.02	55.7	3.54
**4 (AA)**	57.1	3.72	56.1	4.23	56.3	4.04
**Pelvi-acromial index (%)** *Mdn*	63.6^a^	3.29	67.2	3.50	65.9	4.49
**1 (PH)**	64.6	2.49	68.1	2.45	66.7	3.68
**2 (HB, PB, SR)**	64.3	1.44	67.0	4.66	65.0	5.27
**3 (FX, VT)**	65.5	8.02	66.0	2.54	66.0	2.98
**4 (AA)**	60.9	3.15	68.0	3.16	66.5	7.12

^a^ = seniors different from juniors.

1 (PH) = Pommel horse, 2 (HB, PB, SR) = Horizontal bar, parallel bars, rings, 3 (FX, VT) = Floor exercises, vault, 4 (AA) = All-around.

With regard to the lower limb index, no significant interaction was found between age category and preferred event. The effect of age category was significant (F_(1,48)_ = 8.54, p = 0.005, η^2^ = 0.149, Senior<Junior), whereas the effect of preferred event was insignificant. No effect of interaction was observed for trunk-to-limb ratio. We found an effect of age category (F_(1,48)_ = 4.87, p = 0.032, η^2^ = 0.091, Senior>Junior). Furthermore, the effect of preferred event was not significant. No effect of interaction was found for the pelvi-acromial index. However, the effect of the age category was significant (F_(1,48)_ = 15.07, p<0.001, η^2^ = 0.238). There was a significant difference between seniors (mean rank of 17.4) and juniors (mean rank of 33.2). No effect of preferred event was revealed.

### Characteristics of some physical fitness test results according to age category and preferred events

Table 5 shows descriptive statistics of HGSmax, HGS sensitivity, postural balance, CMJ results, and lower limbs power according to age category and preferred events.

**Table 5:** Descriptive statistics of HGSmax, HGS sensitivity, postural balance, results of counter movements jump and lower limbs power for male Polish gymnasts according to age category and preferred events.

Item	Senior		Junior		Total	
**HGSmax (kgf)**	46.6^a^	9.0	31.0	10.9	36.6	12.6
**1 (PH)**	42.4	8.64	31.7	11.7	37.0	11.1
**2 (HB, PB, SR)**	54.0	9.29	30.4	10.4	41.2	15.5
**3 (FX, VT)**	43.5	4.84	37.7	9.12	39.6	8.21
**4 (AA)**	45.2	9.2	27.7	11.0	32.5	13.1
**½ HGSmax (kgf)** *Mdn*	40.0^a^	15.5	19.5	19.0	27.0	26.0
**1 (PH)**	34.8	10.8	24.5	16.5	30.5	11.8
**2 (HB, PB, SR)**	49.0	3.0	12.5	10.0	40.0	36.5
**3 (FX, VT)**	35.5	20.3	26.5	7.75	28.5	12.5
**4 (AA)**	38.5	24.0	15.5	24.8	19.5	31.0
**Rel HGS max (kgf)/kg**	0.68^a^	0.09	0.60	0.10	0.63	0.10
**1 (PH)**	0.60	0.08	0.57	0.17	0.59	0.13
**2 (HB, PB, SR)**	0.76	0.09	0.62	0.12	0.69	0.13
**3 (FX, VT)**	0.66	0.07	0.61	0.09	0.63	0.09
**4 (AA)**	0.66	0.09	0.60	0.08	0.62	0.08
**½ HGS max diff (kgf)**	2.23	8.68	-0.40	5.87	0.54	7.04
**1 (PH)**	0.34	4.63	-1.24	4.27	-0.45	4.21
**2 (HB, PB, SR)**	8.93	5.74	-3.08	5.22	2.38	8.14
**3 (FX, VT)**	-3.10	8.59	-0.92	5.49	-1.65	6.36
**4 (AA)**	1.45	10.8	1.07	6.60	1.18	7.68
**½ HGSmaxdiff (%)**	1.66	9.32	-0.52	8.18	0.26	8.58
**1 (PH)**	0.67	5.30	-0.99	5.63	-0.16	5.14
**2 (HB, PB, SR)**	8.43	5.58	-5.33	7.29	0.93	9.52
**3 (FX, VT)**	-3.74	10.8	-0.63	6.73	-1.66	7.92
**4 (AA)**	0.30	11.4	1.46	9.41	1.14	9.71
**Balance average (s)** *Mdn*	17.11	14.71	17.4	13.36	17.15	13.37
**1 (PH)**	11.05	18.26	25.67	11.84	21.34	21.04
**2 (HB, PB, SR)**	21.65	4.36	15.77	17.64	20.03	15.87
**3 (FX, VT)**	15.86	14.91	13.32	11.07	14.14	12.04
**4 (AA)**	18.53	23.53	17.40	7.57	17.40	11.21
**Balance best (s)**	29.0	13.6	25.9	13.7	27.0	13.6
**1 (PH)**	23.2	18.0	36.8	10.6	30.0	15.5
**2 (HB, PB, SR)**	33.0	12.5	23.9	17.4	28.0	15.4
**3 (FX, VT)**	26.0	14.4	23.5	13.8	24.3	13.4
**4 (AA)**	31.7	12.7	25.1	12.7	26.9	12.7
**CMJ flight time (s)**	0.567^a^	0.032	0.501	0.040	0.525	0.049
**1 (PH)**	0.565	0.034	0.505	0.013	0.535	0.040
**2 (HB, PB, SR)**	0.556	0.031	0.505	0.022	0.528	0.036
**3**^b^ **(FX, VT)**	0.578	0.041	0.541	0.020	0.553	0.032
**4 (AA)**	0.572	0.033	0.477	0.041	0.503	0.058
**CMJ height (cm)**	39.3^a^	4.59	30.9	4.86	33.9	6.24
**1 (PH)**	38.8	4.47	31.2	1.97	35.0	5.17
**2 (HB, PB, SR)**	37.7	4.34	31.3	2.7	34.2	4.71
**3**^c^ **(FX, VT)**	40.9	6.02	35.9	2.68	37.6	4.54
**4 (AA)**	40.0	4.66	28.2	4.9	31.4	7.16
**CMJ (W)** *Mdn*	3538^a^	699.0	2490	1186	2745	1118
**1 (PH)**	3339	1140	2285	439.0	2743	1055
**2 (HB, PB, SR)**	3535	177.0	2280	1461	3039	1610
**3**^d^ **(FX, VT)**	3346	692.5	2928	562.0	3073	652.5
**4 (AA)**	3686	729.0	1580	1813	2574	1707
**CMJ Power (W/kg)**	50.1	4.15	39.9	8.92	43.6	8.99
**1 (PH)**	49.4	3.82	42.4	2.05	45.9	4.73
**2 (HB, PB, SR)**	48.7	4.1	41.6	3.84	44.8	5.29
**3**^e^ **(FX, VT)**	51.8	5.45	47.2	2.36	48.7	4.09
**4 (AA)**	50.7	4.12	35.1	10.5	39.3	11.6

^a^ = seniors different from juniors

^b^ = different from 2 and 4 groups

^c^ = different from 2 and 4 groups

^d^ = different from 1 and 4 groups

^e^ = different from 4 group.

1 (PH) = Pommel horse, 2 (HB, PB, SR) = Horizontal bar, parallel bars, rings, 3 (FX, VT) = Floor exercises, vault, 4 (AA) = All-around.

No interaction was found for HGSmax. An effect of age category was observed (F_(1,48)_ = 26.29, p<0.001, η^2^ = 0.323, Seniors>Juniors), but we found no effect of preferred event. No significant interaction was found for ½HGSmax. The effect of age category (F_(1,48)_ = 12.79, p<0.001, η^2^ = 0.202) was significant. Seniors (mean rank of 36.8) had higher ½HGS max results than juniors (mean rank of 22.2). Similarly, for relative HGSmax, there was no effect of interaction, the effect of age category was significant (F_(1,48)_ = 6.74, p = 0.012, η^2^ = 0.115, Seniors > Juniors), but we found no effect of preferred event.

For difference in ½HGSmax presented in kgf and percentage terms, no significant effect of interaction was found for the age category and preferred event. Two-way ANOVA for the average and best results of the UPST balance test revealed no significant interactions and no main effects for age category and preferred event.

There was no significant interaction for the time of the flight phase during the CMJ without arm swing. Effects were found for the age category (F_(1,48)_ = 43.38, p<0.001, η^2^ = 0.397 (Seniors>Juniors) and preferred event (F_3,48)_ = 4.81, p = 0.005, η^2^ = 0.132, (gr 3> gr 2, gr 3>gr 4). Similarly, no interaction was revealed for the height of CMJ without arm swing. Effects were found for age category (F_1,48)_ = 42.95, p<0.001, η^2^ = 0.398, Seniors > Juniors) and preferred event (F_(3,48)_ = 4.52, p = 0.007, η^2^ = 0.126, gr 3> gr 2, gr 3>gr 4).

No effect of interaction was observed for the power generated during the take-off phase, but we did find effects of age category (F_(1,48)_ = 54.96, p<0,001, η^2^ = 0.261) and preferred event (F_(3,48)_ = 3.96, p = 0.013). There was a significant difference between seniors (mean rank of 41.9) and juniors (mean rank of 19.7). The power in Group 1 (mean rank of 28.1) and Group 4 (mean rank of 26.1) was significantly lower than that in Group 3 (mean rank of 38.6). Furthermore, the relative power (W/kg) did not depend on interaction. Effects were revealed for age category (F_(1,48)_ = 22.810, p<0.001, η^2^ = 0.262, Seniors>Juniors) and preferred event (F_(3,48)_ = 4.23, p = 0.010, η^2^ = 0.146, gr 3 > gr 4).

### A model of athlete characteristics, body build, and fitness components

An optimized PNN model was developed using patterns from experience, mesomorphy, lower limb index, pelvi-acromial index, and relative HGSmax. The model produced an overall prediction accuracy of 77% for discrimination between the best ten male artistic gymnasts and the other 43 participants during the Polish Championships (2017). The optimized PNN model showed similar classifications for the best ten all-around athletes (accuracy 80.0%) and the rest of the group (accuracy 76.7%). The difference of prediction between groups was insignificant (Chi^2^ = 0.000, df = 1, p = 1.000, Cramer’s V = 0.03 [small effect]). The odds ratio between the top-ten group and the rest of the group was 1.212 (Cl = 0.227 to 6.657). This means that the odds ratio of the predicted best ten all-around group to the best ten all-around group is 1.212 times as great as the odds ratio of the predicted rest of the group to the rest of the group.

## Discussion

The main aim of this study was to demonstrate probable differences in training, body build, body composition, and level of motor skills according to age category and preferred gymnastic event. The hypotheses raised in introduction are partially fulfilled. The study used two-way ANOVA to reveal the share of age category and the event preferred by gymnasts in the variance of the measurements. The athletes started their gymnastic training at the same age (≈7 years old), regardless of age category and preferred event. Other studies showed that the mean age when Polish junior gymnasts started training was 6 years [6], whereas that for Olympic medal winners was 7.7 years (and 6.2 years for the period of 1991 to 2000). During the 2012 Summer Olympics in London, the mean age of medal winners was 23.3 years, and the oldest were specialists in SR (27 years). Medium ages were observed for AA athletes (23.7 years), whereas the youngest won medals in the VT event (19.3 years) (4).

When we used the classification of events adopted in our study for the data obtained by Andreev (4), the age of medal winners ranged from 20.8 years (FX, VT) to 25.4 years (HB, PB, SR). In our study, the mean age of seniors was 21.3 years (SD = 2.6), which did not depend on preferred event (from 20.7 to 22.9 year). The differences in age are likely to be caused by the experience necessary to become a specialist in a specific event presented during the Olympic Games.

With regard to experience, we found that senior athletes had two times greater experience than juniors, and there was more experience in Groups 2 (HB, PB, SR) and 3 (FX, FT) than Group 4 (AA). The weekly number of training sessions suggested that seniors who preferred AA trained more (twice per day) than athletes who preferred individual events (once a day). The Russian national team was reported to train three times a day during a centralized preparation period [28]. The factor of age category had an effect on most (22) of the characteristics and indices studied, which can be linked to ontogenesis, susceptibility to training stimuli, and adaptation to training and competitive load. Fewer athletes among seniors and juniors result from the training policy of the Polish Gymnastic Association. However, those who are specialists in all-around events mostly take part in competitions.

A natural predominance seniors over juniors was found in body height and mass. Seniors had a higher mesomorphy component than juniors. Consequently, the senior somatotype (balanced mesomorph) was different from that of juniors (ectomorphic mesomorph). Seniors also had higher BMI, FFM, FM, and FMI compared to juniors, but they did not differ in body fat PF%. No significant differences were found in the anthropometric length indices except for lower limb index, which was as in the range typical of champions (52–56%) in juniors but not in seniors [28].

The pelvi-acromial index was lower in senior athletes (63.55%) than in juniors (67.24%). A group of seniors showed highly consist results with the standards for champions, who are characterized by moderately wide shoulders, narrow hips, long arms, relatively long legs, and a short trunk [28]. A natural consequence of the recruitment for artistic gymnastic teams compared to untrained peers among juniors was lower height (–0.84SD) and body mass (–0.52SD) [30] in seniors (–1.33SD and –0.50SD), respectively) [31].

The BMI in juniors was lower (–0.22SD) but higher in seniors (0.19SD). Compared to non-athletes [31, 32], differences were observed in shoulder width in juniors (–0.05SD) and seniors (0.65SD), whereas pelvis width was lower in juniors (–0.90SD) and seniors (–1.44SD). We found greater values of the pelvi-acromial index in juniors compared to seniors. In junior gymnasts, lower values compared to non-athletes were found for triceps (–0.78SD), subscapular (–0.56SD), abdominal (–0.59SD), and supraspinale (–0.60SD) skinfolds [33]. These differences were even larger in seniors compared to non-athletes for triceps (–1.10SD), subscapular (–0.68SD), abdominal (–0.93SD), and supraspinale (–0.99SD) skinfolds [31]. Due to the specific selection during recruitment for gymnastic teams, gymnasts were characterized by lower height and body mass, greater mesomorphy, and lower body fat compared to non-athletes. Furthermore, higher competitive level was related to greater levels of mesomorphy and FFM [5, 10].

In general, the gymnasts had low fat percentage (9.8 PF%). The individual PF% was 5.7 to 16.2% in seniors and 3.8 to 15.8% in juniors (Fig 4). The fat percentage was in the normal range for each athlete within for their age categories [16]. The structure of the mean results (scores) from individual events in the subgroup of the ten best contemporary Polish all-around athletes was SR > HB > PB > VT > FX > PH. This hierarchy was consist with the classification of events used in our study in Groups 2, 3, and 1. In this context, the comparison of previous results (in points) of all-around events during the Olympic Games was interesting [34].

In the cluster analysis, we identified four patterns of results from six events that were related to total scores for the men’s all-around event. In the best male Olympic athletes, the pattern of scoring No. 1 (based on the judges’ scores) was characterized by the following order: PH > SR > HB > FX > PB > VT. Therefore, the structure of scores obtained by the best all-around athletes was different from those documented for the lower level Olympians and contrasted with the ten best Polish all-around gymnasts. Furthermore, an improvement in the quality of exercises in the most difficult event (with the lowest scores) might leave some room for male contestants to obtain better scores during the gymnastic events [34]. With this in mind, the poorest performance in the group of the ten best Polish all-around gymnasts was found for PH, which represents a distinct indication for improvement in their training practice in preparation for this event. Interestingly, the athletes who specialized in PH and all-around events in our study had homogeneous experience.

Concerning the body build of gymnasts, we found that the athletes from individual competitions did not differ significantly in body weight and height (Table 2). With low and similar body fat, which was also indicated by the value of endomorphy, the athletes who specialized in FX and VT (Group 3) were characterized by low ectomorphy. The PH specialists had the highest ectomorphy. The mesomorphy somatotype component was typical of the gymnasts who preferred PH and was significantly lower than in Group 3 (FX and VT) and Group 2 (HB, PB and SR).

Hand grip is critical to the performance of exercise routines by gymnasts on different apparatuses, such as giant drills or longswings, especially in the downswing phase on horizontal bar. Biomechanics has an impact on handgrip. Loads on the hands of over 13 G have been recorded in certain cases [35]. Therefore, release-regrasp skills are important for HB. We found a natural advantage of the senior age group over juniors in HGSmax, 1/2HGSmax, and HGSmax relative to body mass. In one study [35], HGSmax depended on body mass, but relative strength decreased with increases in absolute strength due to the increase in weight [28]. We concluded that this is likely to be true for the same age category because the comparison between seniors (heavier weight) and juniors (lighter weight) showed similar directions of differences in absolute and relative HGSmax. A comparison between senior gymnasts in the present work and judo athletes (a grasping sport) [12] showed similar results in HGSmax, but gymnasts had higher relative HGSmax (0.68 ± 0.09 kgf/kg) than judokas (0.55 ± 0.11 kgf/kg). Especially in 1/2HGSmax diff (%), gymnasts (1.66 ± 9.32%) demonstrated much higher strength sensitivity than judokas (33.32 ± 22.48%). In another work analyzing combined data for males and females [11], the hand strength relative to HGSmax/body weight was only 0.38.

Keeping balance after dismount from an apparatus and landing is a typical ability needed during both training and competitions, regardless of age category. Although UPST is a non-specific test for gymnasts, the average and best results were similar according to the age category and preferred event. The results among senior and junior gymnasts can be regarded as similar to those obtained for judokas [12]. Longer times (better results) compared to the normative UPST data [23] were found for the average (10.2 ± SE9.6 s) and best times (16.9 ± SE13.9 s). In another experiment, comparison of the average ranks of UPST for the best results with eyes closed revealed significant differences between the three groups (test statistic = 6.71, p = 0.035). A significant difference has been observed between the medians of judokas (15.2 s) and non-athletes (7.9 s) [21].

FX and VT events are characterized by exercises performed in flight, which require a dynamic take-off. This study found that the flight time and height during CMJ, absolute power, and power relative to body mass depend on age category (seniors were superior to juniors in all four measurement results). Another study also found that the flight time and height during CMJ depended on age, with 0.406 s measured for participants aged 21.4 years old and 0.398 s observed or 12-year-old participants. The jump heights were 36.3 cm and 25.2 cm, respectively. The power relative to body mass was 55.91 W/kg in seniors and 41.92 W/kg in the younger group [36]. Better results were obtained by the athletes analyzed in our study.

Suchomel et al. [13] examined artistic gymnasts from the national American junior team (n = 21, age 15.1 ± 1.7 years), who performed extensive gymnastic training (3 to 4 hours a day for 5 days a week). The researchers recorded a mean power of 3636 W for the CMJ jump, which was similar to that obtained by Polish seniors. Kochanowicz et al. [7] analyzed a group of boys aged 9 to 11 years and recorded a mean Pmax of 1401 W. Higher values in the present work were found for slightly older Polish junior gymnasts. In basketball, which is characterized by taller (199.5 ± 8.2 cm) and heavier (96.5 ± 11.2 kg) men than in gymnastics, the vertical jump height was 57.4 ± 7.7 cm, whereas the power generated by the lower limbs was 1582.1 ± 193.6 W [37]. The results for power of the lower limbs in gymnasts (seniors, n = 19, 51.80 W/kg) and basketball players (n = 60, 16.40 W/kg) showed more pronounced intergroup differences, which are related to the demands of both sports.

**Fig 5:**
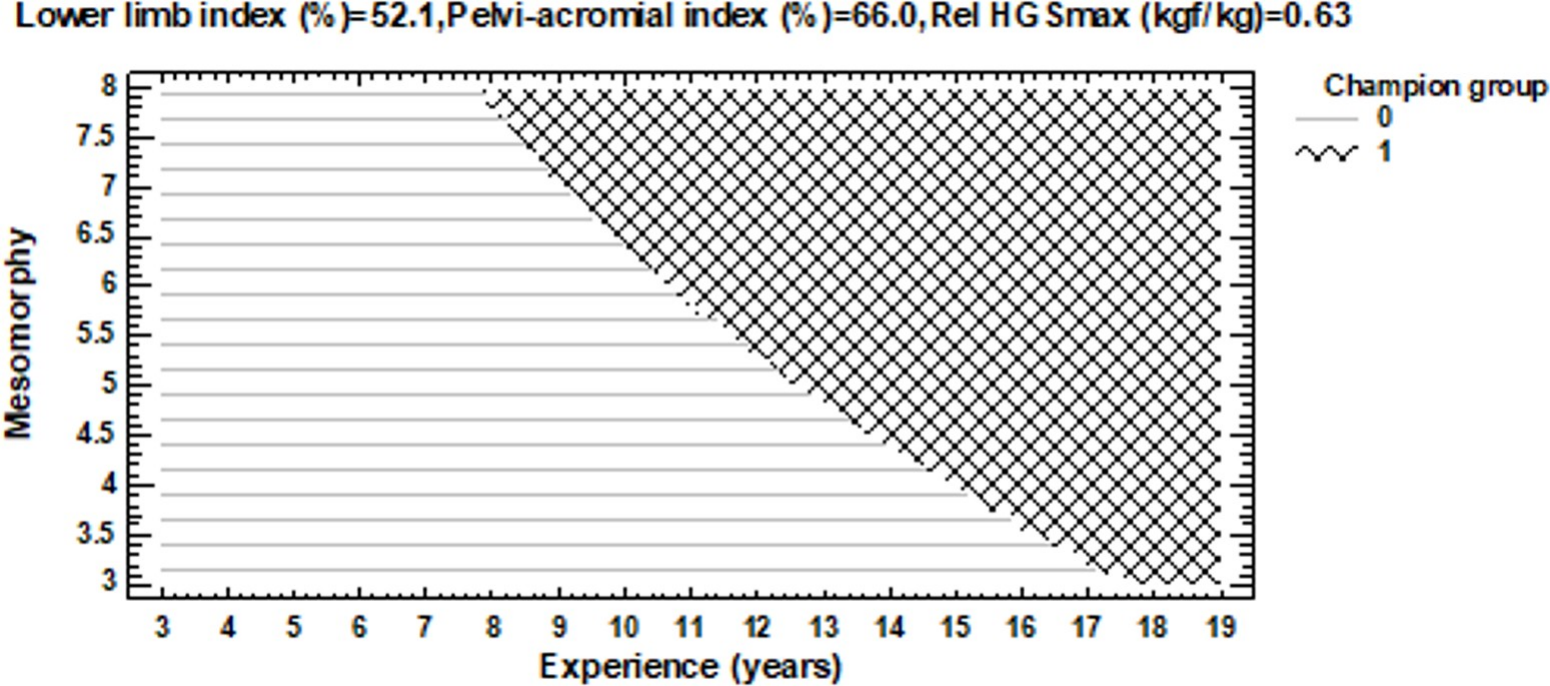
Classification plot. 0—the rest group, 1 –the ten best all-arounders. Each fill type-coded region corresponds to a different group. Experience and mesomorphy are used to define the horizontal and vertical axes, while the lower limb index, pelvi-acromial index, and relative HGSmax are held at fixed values.

The factor of preferred event was related to the results of the tests performed using the Opto-jump device. The groups of preferred events differed in their flight time and CMJ jump height: Group 3 (FX, VT) > Group 2 (HB, PB, SR), Group 3 > Group 4 (AA). The results obtained for CMJ height among seniors were higher than those presented by Dallas et al. [38]. The absolute power in Group 1 (PH) and Group 4 (AA) was significantly lower than in Group 3 (FX and VT). Also, the relative power for Group 3 was better than for Group 4. A reference for a gymnastic event leads to advanced specialization. We also found fitness profiling to be useful for different apparatuses. Based on the results of our study and scores documented for the athletes during the Polish Senior and Junior Artistic Gymnastics Championships (Warsaw 2017), we developed a model of top competitors in gymnastic all-around events using a PNN. The optimized PNN model shows similar classification of the ten best all-around athletes (accuracy 80.0%) and the rest of the group (accuracy 76.7%) using patterns of experience, mesomorphy, lower limb index, pelvi-acromial index, and relative HGSmax (Fig 5).

One strength of this study is its originality. We examined a hard-to-access group of athletes during a competitive period. For the first time, the results of the two-way ANOVA allowed for the evaluation of the strength of effect size (η^2^) of the factor of preferred gymnastic event while considering age category. Particularly notable was the evaluation of the characteristics of body build and motor abilities of outstanding athletes who specialize in individual events. Somatotype, body composition, and fitness characteristics of artistic gymnasts were examined according to depending on age and preferred event. The use of the PNN in examinations of athletes is rather rare [39, 40], but it turned out to be particularly useful in our study. The PNN network was employed to design a model of top athletes of gymnastic events. The optimized PNN model showed similar classification of the ten best all-around athletes and the rest of the group using patterns of experience, mesomorphy, lower limb index, pelvi-acromial index and relative HGSmax. The odds ratio of the predicted best ten all-around group to the rest of the group is low.

A limitation of the study is the relatively low number of people in the subgroups. However, it was impossible to recruit a greater number of artistic gymnasts from Poland for the study. Not all participants of Poland championship were willing to participate in our examinations on the day of competition. Despite these obstacles the gymnasts studied represented the majority of the population of Polish elite gymnasts. Therefore, the findings of the study should be considered as contribution to the state of knowledge concerning variation of the observed phenomena showing a specific pattern of the relationships. We are aware of the fact that only the mesomorphy component and relative HGSmax were sensitive to training in this model. Therefore, in the future, we would like to develop more detailed models of athletes who specialize in various gymnastic events.

## Conclusions

We found the effects of many years of gymnastic training and specific recruitment of athletes on the anthropometric characteristics and indices as well as several motor abilities. These findings allowed for isolation of the independent effects (except for the interaction for FFMI) of age category and preferred gymnastic event on the profile of physical preparation of the athlete. The results revealed the specific characteristics of preparation in the competitive period, and the following conclusions were obtained:

1. Seniors demonstrated natural predominance over juniors in several somatic and fitness variables that was affected by age category of gymnasts. Detected differences can be useful for coaches in the identification and development of gymnastic talent.
2. The detected effect of preferred event on certain variables that characterize body build and physical fitness could be useful for choosing a specialization in a gymnastic event. Those findings should be considered as tendencies and the larger sample of high level gymnast must be examined in the future.
3. The senior athletes that specialize in 1) horizontal bar, parallel bars, rings, 2) all-around were characterized in higher values of FFMI than the rest of sample of Polish gymnasts, that was an effect of interaction between the age category and preferred event.
4. The PNN method demonstrated that a high skill level in all-around events at a national competitive level can be achieved if the athlete is characterized by adequately experience, mesomorphy somatotype component, lower limb index, relative HGSmax, and pelvi-acromial index.

## Supporting information

S1 Data (XLSX)
